# Procalcitonin and C-reactive Protein in Septicaemia: Correlation With Bacterial Profile and Antimicrobial Resistance in ICU Setting

**DOI:** 10.7759/cureus.112605

**Published:** 2026-07-13

**Authors:** Vishakha S Pawar, Ravindra V Shinde, Satish Patil

**Affiliations:** 1 Department of Microbiology, Krishna Vishwa Vidyapeeth (Deemed To Be University), Karad, IND

**Keywords:** antibiotic stewardship (ams), antimicrobial resistance, bacterial infection, c-reactive protein, intensive care unit (icu), procalcitonin, septicaemia, serum biomarkers

## Abstract

Septicemia remains a major cause of morbidity and mortality among critically ill patients admitted to intensive care units (ICUs), particularly in regions facing a growing burden of antimicrobial resistance. Early diagnosis and prompt initiation of appropriate antimicrobial therapy are essential for improving patient outcomes; however, conventional blood culture methods often require prolonged turnaround times, limiting their utility in urgent clinical decision-making. This narrative review aims to summarize current evidence regarding the epidemiology, microbiological characteristics, antimicrobial resistance patterns, and clinical utility of inflammatory biomarkers in ICU-associated septicemia, with particular emphasis on C-reactive protein (CRP) and procalcitonin (PCT). Relevant peer-reviewed literature was identified through a review of published studies, reviews, and clinical guidelines addressing septicemia, biomarker performance, antimicrobial resistance, and antimicrobial stewardship in critical care settings. Available evidence suggests that gram-negative pathogens remain predominant causes of ICU septicemia and are frequently associated with elevated PCT concentrations due to endotoxin-mediated inflammatory responses. Compared with CRP, PCT generally demonstrates earlier elevation following bacterial infection, greater specificity for bacterial sepsis, and a closer association with disease severity. However, both biomarkers have limitations, particularly in predicting mortality and clinical outcomes. Current evidence supports the use of biomarkers as adjunctive tools rather than standalone diagnostic tests. PCT-guided antimicrobial strategies may contribute to improved antimicrobial stewardship and reduced unnecessary antibiotic exposure in selected patient populations. Overall, optimal management of septicemia requires integration of clinical assessment, microbiological investigations, and biomarker data to support timely and informed therapeutic decisions.

## Introduction and background

Historical background

Sepsis has been recognized in medical literature for more than two millennia. The term originates from ancient Greek texts, where it was used to describe decay or putrefaction. Early physicians, including Hippocrates and Galen, recognized sepsis as a serious clinical condition associated with progressive deterioration and death, although its underlying mechanisms remained poorly understood [1].

The modern understanding of sepsis began to emerge in the early 20th century when Hugo Schottmüller linked the condition to microbial invasion of the bloodstream and systemic illness [2]. Subsequent efforts to standardize diagnosis led to the development of the Sepsis-1 criteria in 1991, which defined sepsis as infection accompanied by a systemic inflammatory response [3]. Later revisions expanded the diagnostic framework, culminating in the Sepsis-3 definition, which characterizes sepsis as a life-threatening organ dysfunction caused by a dysregulated host response to infection [4,5].

Alongside the evolution of sepsis definitions, considerable attention was directed toward identifying biomarkers capable of facilitating earlier diagnosis. C-reactive protein (CRP), an acute-phase reactant, was recognized as a marker of inflammation in the early twentieth century [6]. Procalcitonin (PCT), initially identified as a precursor of calcitonin, gained clinical significance in 1993 when elevated serum levels were shown to be strongly associated with bacterial infections and sepsis [7].

Despite advances in sepsis definitions and management, early diagnosis remains a major challenge in critical care medicine. Clinical manifestations are often nonspecific and may overlap with non-infectious inflammatory conditions, making accurate diagnosis difficult. Because delayed recognition and treatment are associated with increased morbidity and mortality, there has been growing interest in reliable biomarkers that can support early diagnosis and therapeutic decision-making. Among the numerous biomarkers studied, CRP and PCT have emerged as the most widely used and clinically relevant indicators in sepsis management.

Introduction

Septicaemia remains a major cause of morbidity and mortality among critically ill patients admitted to intensive care units (ICUs). Early recognition and prompt initiation of appropriate antimicrobial therapy are essential for improving patient outcomes, reducing hospital stay, and minimizing healthcare costs [8].

However, distinguishing bacterial sepsis from viral infections or non-infectious systemic inflammatory conditions remains challenging because of overlapping clinical features. Conventional blood culture techniques, although considered the diagnostic gold standard, often require two to five days to produce clinically actionable results and may yield false-negative findings in patients with intermittent bacteremia or prior antibiotic exposure [9,10].

To overcome these limitations, clinicians increasingly rely on biomarkers that reflect the host inflammatory response to infection. Among the available biomarkers, CRP and PCT are the most extensively studied and commonly used in clinical practice. CRP is a readily available marker of systemic inflammation but lacks specificity for bacterial infection. In contrast, PCT demonstrates more rapid kinetics and greater specificity for bacterial sepsis, making it a valuable adjunct in diagnosis, prognostication, and antimicrobial stewardship [11,12].

Despite their widespread clinical use, uncertainty remains regarding the relative diagnostic accuracy, prognostic value, and optimal application of CRP and PCT in critically ill patients with suspected septicemia. This narrative review aims to compare the biological characteristics, diagnostic performance, clinical utility, and limitations of CRP and PCT in the diagnosis, prognosis, and management of septicemia in intensive care settings.

Methods

Relevant literature was identified through searches of PubMed, Scopus, Google Scholar, and Web of Science databases. Search terms included "sepsis", "septicaemia", "intensive care unit", "procalcitonin", "C-reactive protein", "biomarkers", "bloodstream infection", "antimicrobial resistance", and "antimicrobial stewardship". Original articles, systematic reviews, meta-analyses, and clinical guidelines published in English were considered. Studies unrelated to sepsis biomarkers or critical care were excluded. The retrieved literature was screened for relevance to the objectives of the review, and studies considered most pertinent to the diagnostic and prognostic utility of CRP and PCT in septicemia were included in the final narrative synthesis. As this is a narrative review, no formal risk-of-bias assessment, evidence grading, or meta-analysis was performed.

## Review

Brief scenario

Several studies have evaluated the diagnostic relevance of PCT and CRP in patients with suspected sepsis. PCT was identified as a biomarker of bacterial sepsis and became one of the most extensively studied biomarkers for the diagnosis and prognosis of sepsis [7].

CRP is an acute-phase protein synthesized by the liver in response to inflammation; however, its clinical utility is limited by delayed elevation and poor specificity, as increased levels may occur in trauma, surgery, and other non-infectious conditions [13]. In contrast, PCT rises more rapidly during bacterial infections and demonstrates greater specificity because its production is stimulated by bacterial toxins and inflammatory cytokines while being suppressed during most viral infections [12,14].

Meta-analyses have reported superior diagnostic performance of PCT compared with CRP, with higher sensitivity, specificity, and area under the receiver operating characteristic curve (AUROC) values for the diagnosis of bacterial sepsis. Furthermore, serial PCT measurements may assist in monitoring treatment response and guiding antibiotic stewardship strategies. Nevertheless, neither biomarker should be used in isolation, and both should be interpreted alongside clinical assessment and microbiological findings [15,16].

Epidemiology of septicaemia in the ICU setting

As a primary cause of morbidity and mortality in critically sick patients, sepsis imposes a substantial burden on global healthcare systems. It is estimated to affect nearly 49 million people annually, resulting in approximately 11 million deaths worldwide [17]. The absolute burden is still quite high because of population increase and aging; even though in the United States, in-hospital mortality from sepsis declined from 27.8% to 17.9% between 1979 and 2000, the absolute number of sepsis-related deaths increased. Patients with severe sepsis and septic shock involving three or more failing organs had approximately 70% mortality [18]. 

The worldwide burden varies significantly, especially in low- and middle-income countries where delayed diagnosis and restricted access to organ assistance are common. Less developed nations account for almost 90% of global mortality from meningitis, pneumonia, and other illnesses. Malnutrition, low immunisation rates, and inadequate hygiene standards significantly increase the epidemiological load in low-resource settings [19]. International audits verify that recurrence rates and results of sepsis vary greatly between continents. For instance, research evaluating ICUs around the world revealed that hospital mortality rates for sepsis patients ranged from 11.9% in Oceania to 39.5% in Africa [20].

These global variations are further reflected in clinical classification frameworks, where standardized sepsis definitions incorporate a range of clinical, inflammatory, hemodynamic, and organ dysfunction parameters to assess disease severity across different healthcare settings [21]. Table 1 was compiled from the Sepsis-3 consensus definitions and related critical care guidelines and summarises key clinical and laboratory indicators used to assess sepsis severity.

**Table 1:** Clinical and laboratory parameters used for assessment of sepsis severity and organ dysfunction according to contemporary sepsis criteria SBP: systolic blood pressure; MAP: mean arterial pressure; PaO₂/FiO₂: arterial oxygen partial pressure/fraction of inspired oxygen ratio; INR: international normalized ratio; aPTT: activated partial thromboplastin time; CRP: C-reactive protein; PCT: procalcitonin Reference: [21]

Category	Parameters
Clinical Indicators	Fever (>38°C) or hypothermia (<36°C), altered mental status, tachypnoea
Cardiovascular Dysfunction	Systolic blood pressure <90 mmHg, mean arterial pressure <65 mmHg, requirement for vasopressors
Respiratory Dysfunction	PaO₂/FiO₂ ratio <300, need for mechanical ventilation
Renal Dysfunction	Oliguria, elevated serum creatinine
Hematological Dysfunction	Thrombocytopenia, coagulation abnormalities (INR, aPTT)
Hepatic Dysfunction	Hyperbilirubinemia
Tissue Perfusion Abnormalities	Elevated serum lactate, delayed capillary refill, mottling
Inflammatory Biomarkers	Elevated C-reactive protein (CRP), elevated procalcitonin (PCT), leukocytosis or leukopenia

Bacterial profile of septicaemia

A variety of infections, usually categorized as nosocomial or community-acquired, are involved in the specific etiology of septicemia. Patients in ICUs are especially susceptible to bacterial sepsis because of immune system dysregulation, frequent exposure to invasive procedures, including central venous catheters and mechanical ventilation, and extended hospital stays [3,22]. In addition to microbial distribution, biomarker-guided strategies have been explored in intensive care settings, where PCT-based algorithms have demonstrated effectiveness in optimizing antibiotic use without compromising clinical outcomes [23]. Although both gram-positive and gram-negative bacteria are significant contributors to sepsis, their geographic distribution differs. In 2020, global data showed that while gram-positive bacteria were more common in North America, making up 46% of isolates, gram-negative bacteria were more common in Eastern Europe (78%), Africa (78%), Asia, and the Middle East (76%) [20]. Knowledge of the prevailing bacterial profile in individual healthcare settings is essential for selecting appropriate empirical antimicrobial therapy in patients with suspected septicaemia [24].

Gram-Negative Organisms

The worldwide Intensive Care over Nations (ICON) audit demonstrated that gram-negative organisms constitute the majority of microbiologically documented infections in ICU patients with sepsis, with *Escherichia coli*, *Klebsiella* spp., *Pseudomonas aeruginosa*, and *Acinetobacter* spp. among the predominant pathogens. The spectrum of causative organisms continues to evolve and varies across healthcare settings and geographic regions [20,25].

*P. aeruginosa* and *Klebsiella pneumoniae* were frequently shown to be the primary causal agents in studies assessing extended stays in ICUs. Due to their high levels of resistance to carbapenems and broad-spectrum cephalosporins, these organisms pose significant problems and are highly suited to survive on clinical equipment [26].

Gram-Positive Organisms

A large percentage of bloodstream infections are caused by gram-positive bacteria, which are still common in hospital-acquired illnesses. Methicillin-resistant *Staphylococcus aureus* and coagulase-negative *Staphylococcus* are the most prevalent gram-positive organisms that can cause bloodstream infections when connected to indwelling devices [27].

Gram-positive pathogens commonly implicated in bloodstream infections include coagulase-negative staphylococci, *S. aureus*, *Streptococcus pneumoniae*, and *Enterococcus* spp., with coagulase-negative staphylococci representing the most frequently isolated gram-positive organism in the evaluated blood cultures. Rapid molecular identification of these pathogens facilitates earlier targeted antimicrobial therapy and improves clinical decision-making [11,28].

*Enterococcus* spp., including *Enterococcus faecium*, are important bloodstream pathogens. Rapid laboratory identification of these organisms enables earlier targeted antimicrobial therapy, which has been associated with improved patient outcomes, reduced mortality, decreased hospital length of stay, and optimized antimicrobial utilization [29].

Antibiotic resistance in septicaemia 

The increasing prevalence of antimicrobial resistance has complicated the selection of effective empirical antibiotic therapy in patients with sepsis. Prompt initiation of appropriate antimicrobial treatment remains essential, as delays in administering effective antibiotics are independently associated with increased mortality in septic shock [30]. Antimicrobial resistance poses a major challenge in the empirical management of sepsis. The Surviving Sepsis Campaign recommends that empirical antimicrobial therapy be guided by local antibiograms, patient-specific risk factors for multidrug-resistant pathogens, and subsequent de-escalation based on microbiological results and clinical response [31].

Rhodes et al. summarized contemporary evidence-based recommendations for antimicrobial management in sepsis, emphasizing early administration of broad-spectrum antibiotics, assessment of multidrug-resistant pathogen risk, timely de-escalation, and antimicrobial stewardship to optimize patient outcomes and minimize the emergence of antimicrobial resistance as described in Table 2 [31].

**Table 2:** Key recommendations for antimicrobial therapy and antimicrobial stewardship in adult patients with sepsis and septic shock MDR: multidrug-resistant; MRSA: methicillin-resistant *Staphylococcus aureus*; PCT: procalcitonin; IV: intravenous. Reference:  [31]

Resistance issue	Guideline recommendation
Increasing MDR pathogens	Choose empirical therapy according to local antibiogram and patient-specific MDR risk factors
Previous antibiotic use	Consider when selecting empirical antibiotics
Previous MDR colonization/infection	Broaden empirical coverage accordingly
MRSA risk	Include anti-MRSA therapy
MDR Gram-negative risk	Consider combination therapy with two antimicrobial classes
De-escalation	Narrow therapy once cultures identify the pathogen
Stewardship	Daily reassessment to minimize unnecessary antimicrobial exposure

Pathophysiology of sepsis and biomarker response

Sepsis is characterized by an uncontrolled and dysregulated immune response to infection. The initiation of this host response occurs when the immune response is triggered when microbial components interact with host immune receptors, such as the Lipid A component of lipopolysaccharides in gram-negative organisms or lipoteichoic acid in gram-positive bacteria, binding to specific pattern recognition receptors on host innate immune cells [3].

These receptors, which comprise cytoplasmic nucleotide-binding oligomerization domain (NOD)-like receptors and surface Toll-like receptors, cause macrophages and monocytes to release large amounts of proinflammatory cytokines such as interleukin (IL)-1, IL-6, and tumour necrosis factor-alpha (TNF-α). Leukocytes, the complement system, and hepatic acute-phase reactants are all activated by the ensuing systemic inflammation, which eventually results in severe tissue perfusion deficits and cell death [2].

Based on origin and induction kinetics, biological markers' reactions to this pathophysiological process differ considerably. CRP, an acute-phase protein, is mostly produced by the liver in response to controlled cytokines such as IL-6. It works by attaching itself to phosphocholine on bacteria and dead or dying cells, which triggers the complement system and encourages phagocytosis. CRP rises gradually and peaks about 36 hours after endotoxin challenge due to its sluggish production mechanism [6].

The thyroid gland's C cells typically produce PCT, the precursor protein of calcitonin. Massive amounts of PCT are released into the bloodstream by numerous tissues such as the liver, lungs, intestine, and leukocytes throughout the body during bacterial infection, which is caused by both direct and indirect bacterial and inflammatory mediator-induced PCT production. On the other hand, PCT production is inhibited by cytokines like interferon-gamma that are generated as a result of viral infections. In contrast to viral or non-infectious inflammatory situations, this enables PCT to function as a highly specific biomarker of bacterial aetiology [32].

Bacterial exotoxins contribute significantly to the elevation of procalcitonin levels during sepsis. Toxins produced by organisms such as *S. aureus *and *Streptococcus pyogenes*, particularly superantigens, are capable of activating a large population of T lymphocytes in a non-specific manner. This widespread immune activation results in an increased release of proinflammatory cytokines, including IL-1, IL-6, and TNF-α, which play a central role in stimulating PCT production. Along with this indirect effect, certain bacterial toxins may also directly influence host tissues by enhancing calcitonin gene expression in organs such as the liver and lungs. The combined effect of exotoxins and endotoxins amplifies the systemic inflammatory response, leading to increased procalcitonin release from multiple tissues. This mechanism accounts for the marked rise in procalcitonin observed in bacterial sepsis and supports its usefulness in differentiating bacterial infections from viral or non-infectious inflammatory conditions [2,12].

Common resistance mechanisms

Bacterial pathogens encountered in ICUs employ both enzymatic and non-enzymatic mechanisms to evade antimicrobial agents. Enzymatic resistance is primarily mediated by antibiotic-inactivating enzymes, including β-lactamases and aminoglycoside-modifying enzymes. β-lactamases are classified according to the Ambler molecular classification (classes A-D), with extended-spectrum β-lactamases (ESBLs), predominantly belonging to class A, hydrolysing extended-spectrum cephalosporins, while carbapenemases have emerged as one of the most clinically important mechanisms limiting therapeutic options against multidrug-resistant gram-negative organisms [33].

Non-enzymatic resistance mechanisms further reduce antimicrobial efficacy by decreasing intracellular drug concentrations through reduced outer membrane permeability, including porin alterations such as OprD loss, and by increasing the activity of bacterial efflux systems that actively expel antimicrobial agents. In addition, modification of antimicrobial target sites reduces antibiotic binding, while horizontal gene transfer through conjugation, transformation, and transduction facilitates the dissemination of resistance determinants among bacterial species, contributing substantially to the spread of multidrug resistance in hospital environments [33].

Laboratory diagnosis of septicaemia

Improving patient outcomes and preventing unnecessary exposure of uninfected individuals to broad-spectrum antibiotics depend on the rapid and accurate diagnosis of septicaemia. Because the clinical manifestations of sepsis are often non-specific, clinicians rely heavily on laboratory investigations to confirm the diagnosis, identify causative pathogens, and guide targeted antimicrobial therapy [8,31].

Several laboratory techniques are available for the estimation of serum procalcitonin, including enzyme-linked immunosorbent assay (ELISA), enzyme-linked fluorescent assay (ELFA), chemiluminescent immunoassays (CLIA), time-resolved fluoroimmunoassays (TRFIA), immunochromatographic tests (ICT), and immunoluminometric assays. Automated immunoassay platforms provide rapid, sensitive, and reliable quantification of serum PCT, making them particularly valuable in intensive care settings [34].

Recent advances in rapid diagnostic technologies, including automated microbial identification systems and molecular diagnostic methods, have substantially improved the speed and accuracy of pathogen detection in bloodstream infections, enabling earlier targeted antimicrobial therapy and supporting antimicrobial stewardship programmes [8,31].

Clinical application and antibiotic stewardship

Numerous clinical studies have demonstrated the value of PCT in guiding antimicrobial therapy for patients with sepsis. Early recognition of bacterial infection and prompt initiation of appropriate antimicrobial therapy are critical, as delays in effective antibiotic treatment are independently associated with increased mortality in severe sepsis. At the same time, antimicrobial stewardship remains essential to minimize unnecessary antibiotic exposure, reduce the emergence of multidrug-resistant organisms, and optimize clinical outcomes [8,30,31].

PCT-guided treatment algorithms support decisions regarding antibiotic continuation and discontinuation based on serial serum PCT measurements in conjunction with clinical assessment. Several studies have shown that PCT-guided strategies safely reduce antibiotic exposure and treatment duration without increasing mortality, while supporting antimicrobial stewardship efforts [15,31].

Limitations

Despite its clinical utility, PCT has several important limitations. Elevated PCT concentrations may occur in certain non-infectious conditions, including major trauma, extensive surgery, severe burns, and cardiogenic shock, thereby reducing its specificity for bacterial infection. In addition, physiological elevations during the neonatal period may complicate interpretation in early-onset neonatal sepsis. PCT testing is also more expensive than CRP, which may limit its routine use, particularly in resource-limited healthcare settings [6,11,13].

Furthermore, biomarkers should not be interpreted in isolation but rather alongside clinical findings, microbiological investigations, and other laboratory parameters to improve diagnostic accuracy and guide patient management [7,15].

Future directions

Future improvements in sepsis management are expected to arise from advances in rapid diagnostic technologies that enable earlier pathogen identification and facilitate timely targeted antimicrobial therapy, thereby supporting antimicrobial stewardship programmes [8].

Biomarker-guided antimicrobial therapy continues to evolve as an important strategy for optimizing antibiotic use while reducing unnecessary antimicrobial exposure. Current evidence also emphasizes that biomarkers should be interpreted alongside clinical findings and microbiological investigations rather than in isolation to improve diagnostic accuracy and patient management [7,31].

Continued research is needed to further refine the diagnostic and prognostic utility of sepsis biomarkers and to integrate them more effectively into routine clinical practice, with the aim of improving individualized patient care and clinical outcomes [7].

## Conclusions

High mortality rates and the increasing prevalence of multidrug-resistant organisms continue to make septicemia a major challenge in ICUs. Although microbiological cultures remain the gold standard for definitive diagnosis, their prolonged turnaround time necessitates the use of biomarkers to support early diagnosis and clinical monitoring. PCT generally demonstrates more favorable kinetic characteristics and greater specificity for bacterial infections than CRP, making it a valuable adjunct in the evaluation of patients with suspected sepsis. Furthermore, PCT-guided strategies have shown potential in supporting antimicrobial stewardship efforts by reducing unnecessary antibiotic exposure in selected patient populations.

However, neither biomarker should be used as a standalone diagnostic tool. Both PCT and CRP should be interpreted in conjunction with clinical assessment, microbiological findings, local antimicrobial resistance patterns, and other laboratory parameters. This review highlights the complementary roles of these biomarkers in the diagnosis and management of septicemia in intensive care settings and underscores the importance of an integrated approach to improve clinical decision-making and patient outcomes. 
